# Supplementary material for: An atypical mating system in a neotropical manakin
Source: R Soc Open Sci. 2020 Jan 8;7(1):191548. doi: 10.1098/rsos.191548 (PMC7029923; doi:10.1098/rsos.191548)
Supplement: Genotyping and paternity analyses: Table1 and Table 2 [file rsos191548supp1.docx]

**SUPPLEMENTARY MATERIAL**

From the manuscript: Selection for an Atypical Mating System in a Neotropical Manakin.

Milene G. Gaiotti, Michael S. Webster, and Regina H. Macedo

**Genotyping and paternity analyses**

DNA was extracted using a standard extraction protocol (QIAGEN®) and sexed adults and nestlings using the primer set 2550F/2718R [1]. We genotyped samples from 96 chicks from 60 nests using 15 polymorphic microsatellite markers [2-5] (Tables S1 and S2) and ran paternity analysis in Cervus 3.0.0 [6]. Ten of the microsatellite loci designed for other species were optimized for Araripe manakins and used to determine parentage. Five primers were developed specifically for *A. bokermanni* [4]. There were 67 alleles in the 15 loci, ranging from 2 to 8 with an average of 4.5 per locus (Table S1 and S2). We assigned parentage to the most likely candidate parents under relaxed (90%) and strict (95%) levels of confidence, by calculating the likelihood ratio scores (with the single exception of one male with the highest LOD, which defended the territory where the brood hatched, where an 80% level of confidence was accepted). Critical values of these scores were estimated through simulations in Cervus with the following parameters: (i) 10000 simulated nestling genotypes; (ii) number of candidate fathers; (iii) proportion of loci typed; (iv) proportion of males sampled in the population (85%); and (v) minimum number of desired alleles compatible between fathers and nestlings (modified to 7). Maternity analyses confirmed that all nesting females were the biological mothers of broods they were caring for, although we used the parameter of 70% for proportion of females sampled in the population. Simulations for paternity were made for nestlings where maternity was determined through field observations as well as when maternity was unknown. Paternity was only confirmed for a nestling when: (1) the LOD score for the trio mother-nestling-father was higher than 6.45 (i.e. more than 95% confidence by Cervus; [6]); (2) the confirmed mother´s LOD score was higher than 4.32; (3) the number of mismatching alleles between nestling and father was less than 2; and (4) in the few cases of very close LOD scores (N = 5), the male captured at the nearest distance to the nest was considered the father. To further clarify our understanding of paternity patterns taking into consideration genetic maternity, we generated figures for the LOD score trios to compare the scores and mismatches between nestlings and potential fathers using (1) males that defended territories within which broods hatched; and (2) using biological fathers.

**Table S1.** Description of 15 microsatellite loci used for the Araripe manakin analyses, their respective annealing temperatures (Ta) and source species.

| **Locus** | **Primer sequence (5'-3')** | **Ta (°C)** | **Source species** |
| --- | --- | --- | --- |
| Chiro10 | F:CGAGTTTTCCCAGTCACGACGGAGGGATAGTGGGCAAGAT | 51 | *Chiroxiphia lanceolata* [5] |
|  | R:CTTTCTGGAGTTCTTTCCATGC |  |  |
| Chiro4 | F:CGAGTTTTCCCAGTCACGACACACTGGCTACACCAGTCAATC | 51 | *Chiroxiphia lanceolata* [5] |
|  | R:ATTGCATTCTTCACTGCTCAAG |  |  |
| Chiro5 | F:CGAGTTTTCCCAGTCACGACTGAGAGGCAGAAAATATTCCAG | 51 | *Chiroxiphia lanceolata* [5] |
|  | R:GATTAGATCTTCATCCTGAGAGT |  |  |
| Chiro8 | F:CGAGTTTTCCCAGTCACGACTTGTACAGACAATCACATCTACCTC | 51 | *Chiroxiphia lanceolata* [5] |
|  | R:TGACAATGTGGGTGTATGCAG |  |  |
| Chiro7 | F:CGAGTTTTCCCAGTCACGACTCACCCTTTCAGTTCTTTCTCC | 58 | *Chiroxiphia lanceolata* [5] |
|  | R:CACTGTCACATGCGTTTCATTAG |  |  |
| Chiro11 | F:CGAGTTTTCCCAGTCACGACACAGCAAATGACTGTCAAGAGG | 58 | *Chiroxiphia lanceolata* [5] |
|  | R:TGCACTAGCTGATGCTTACTGA |  |  |
| Chiro12 | F:CGAGTTTTCCCAGTCACGACTGTCCCTTATTTGCCTTAGTGC | 58 | *Chiroxiphia lanceolata* [5] |
|  | R:GCTTCACTTGCAATACATGTCTATCT |  |  |
| Man1 | F:CGAGTTTTCCCAGTCACGACCAGCATCTATCTGTCTATCTGTCTG | 58 | *Manacus manacus* [2] |
|  | R:CGACAGGATAAACTCAAAAAGC |  |  |
| Man4 | F:CGAGTTTTCCCAGTCACGACAGCATCTATCTGTCTATCTGTCTG | 58 | *Manacus manacus* [2] |
|  | R:GAGGAAAATAAAGCCCAGAAG |  |  |
| Chir3-22 | F:TGAAGTCCAGAGACAACAG | 58 | *Chiroxiphia caudata* [3] |
|  | R:GAACTAATGCAACTTCTGAG |  |  |
| Abom7 | F:TTTTCCCAGTCACGACACCCATTGCTATTGGGCAAC | 53 | *Antilophia bokermanni* [4] |
|  | R:CAGGACTGCCTTTTAGATGAGG |  |  |
| Abom9 | F:TTTTCCCAGTCACGACGAGGAAGAGGAAGGGAAAAAGA | 55 | *Antilophia bokermanni* [4] |
|  | R:AACAAGTCCCGTTCTCATGC |  |  |
| Abom10 | F:TTTTCCCAGTCACGACATTCTGGCTCAAGCAAAAGC | 55 | *Antilophia bokermanni* [4] |
|  | R:AACGTGCTAGAGTGGGTTGC |  |  |
| Abom12 | F:TTTTCCCAGTCACGACTGCAAGGATGGACTGACTCTT | 55 | *Antilophia bokermanni* [4] |
|  | R:CACTTTTTCAAGGGTTTCAAAT |  |  |
| Abom14 | F: TTTTCCCAGTCACGACCTCATGCTAACTCAGCTGAC | 53 | *Antilophia bokermanni* [4] |
|  | R:GGGAAAAAGAAGAAAATATGGAAGA |  |  |

**Table S2.** Characteristics of 15 microsatellite loci amplified for 359 *Antilophia bokermanni* individuals sampled and used in maternity and paternity analyses. Hobs: observed heterozygosity; Hexp: expected heterozygosity; NE-1P: exclusion probability for one candidate parent; NE-2P: exclusion probability for one candidate father given the genotype of the mother (for offspring with confirmed maternity); P(HW): probability of Hardy-Weinberg equilibrium; and FN: frequency of null alleles.

| **Locus** | **No. alleles** | **Hobs** | **Hexp** | **NE-1P** | **NE-2P** | **P (HW)** | **FN** |
| --- | --- | --- | --- | --- | --- | --- | --- |
| Chiro5 | 4 | 0.621 | 0.564 | 0.833 | 0.677 | 0.002 | -0.0550 |
| Chiro4 | 8 | 0.710 | 0.726 | 0.673 | 0.492 | 0.410 | 0.0153 |
| Chiro10 | 5 | 0.474 | 0.447 | 0.895 | 0.754 | 0.340 | -0.0290 |
| Chiro8 | 4 | 0.630 | 0.532 | 0.851 | 0.697 | 0.000 | -0.1039 |
| Chiro12 | 5 | 0.643 | 0.635 | 0.780 | 0.623 | 0.5901 | -0.0089 |
| Chiro11 | 3 | 0.599 | 0.570 | 0.838 | 0.725 | 0.6339 | -0.0239 |
| Man4 | 6 | 0.691 | 0.702 | 0.709 | 0.532 | 0.7368 | 0.0066 |
| Man1 | 6 | 0.727 | 0.710 | 0.699 | 0.521 | 0.3197 | -0.0160 |
| Chr3-22 | 3 | 0.284 | 0.295 | 0.956 | 0.864 | 0.8266 | 0.0128 |
| Chiro7 | 7 | 0.730 | 0.728 | 0.675 | 0.497 | 0.006 | -0.0023 |
| Abom 7 | 2 | 0.485 | 0.497 | 0.877 | 0.813 | 0.6966 | 0.0118 |
| Abom 9 | 5 | 0.752 | 0.731 | 0.685 | 0.512 | 0.5319 | -0.0152 |
| Abom 10 | 2 | 0.382 | 0.404 | 0.919 | 0.839 | 0.3497 | 0.0274 |
| Abom 12 | 2 | 0.474 | 0.461 | 0.894 | 0.823 | 0.6702 | -0.0140 |
| Abom 14 | 5 | 0.747 | 0.732 | 0.684 | 0.510 | 0.6940 | -0.0107 |

**References for Supplemental Materials**

1. Griffiths, R., Daan, S., and Dijkstra, C. (1996). Sex identification in birds using two CHD genes. Proc. R. Soc. B. Biol. 263, 1251-1256.
<https://doi.org/10.1098/rspb.1996.0184>

2. Piertney, S.B., Shorey, L., and Höglund, J. (2002). Characterization of microsatellite DNA markers in the white-bearded manakin (Manacus manacus). Mol. Ecol. Notes 2, 504-505.
<https://doi.org/10.1046/j.1471-8286.2002.00292.x>

3. Francisco, M.R., Galetti, P.M., and Gibbs, H.L. (2004). Isolation and characterization of microsatellite loci in the blue manakin, Chiroxiphia caudata (Aves, Pipridae). Mol. Ecol. Notes. 4, 758-760.
<https://doi.org/10.1111/j.1471-8286.2004.00812.x>

4. Souza, T.O., Luna, L.W., Melo, M.A.D., Araripe, J., and Rêgo, P.S. 2019. Characterization of the genetic diversity and population structure of the manakin genus Antilophia through the development and analysis of microsatellite markers. J. Ornithol. Doi.org/10.1007/s10336-019-01655-w
<https://doi.org/10.1007/s10336-019-01655-w>

5. Duval, E.H., Carter, K.L., and Kempenaers, B. (2007). Isolation and characterization of novel microsatellite loci for parentage assessment in the lance-tailed manakin (Chiroxiphia lanceolata). Mol. Ecol. Notes 7, 1111-1113.
<https://doi.org/10.1111/j.1471-8286.2007.01795.x>

6. Kalinowski, S.T., Taper, M.L., and Marshall, T.C. (2007). Revising how the computer program CERVUS accommodates genotyping error increases success in paternity assignment. Mol. Ecol. 16, 1099-1106.
<https://doi.org/10.1111/j.1365-294X.2007.03089.x>
